# MHCII-restricted T helper cells: an emerging trigger for chronic tactile allodynia after nerve injuries

**DOI:** 10.1186/s12974-019-1684-0

**Published:** 2020-01-03

**Authors:** You-Quan Ding, Han Luo, Jian-Guo Qi

**Affiliations:** 1 Department of Histology, Embryology and Neurobiology, West China School of Basic Medical Sciences and Forensic Medicine, Sichuan University, No 17, Section 3, South Ren-min road, Chengdu, 610041 Sichuan China; 2 Department of Thyroid and Parathyroid Surgery, National Clinical Research Center for Geriatrics, West China Hospital, Sichuan University, Chengdu, Sichuan China

**Keywords:** Nerve injury, Neuropathic pain, Chronic pain, Tactile allodynia, Th cell, MHCII, T cell, Neuroimmunology, Dorsal root leptomeninge, Precision medicine

## Abstract

Nerve injury-induced chronic pain has been an urgent problem for both public health and clinical practice. While transition to chronic pain is not an inevitable consequence of nerve injuries, the susceptibility/resilience factors and mechanisms for chronic neuropathic pain after nerve injuries still remain unknown. Current preclinical and clinical studies, with certain notable limitations, have shown that major histocompatibility complex class II–restricted T helper (Th) cells is an important trigger for nerve injury-induced chronic tactile allodynia, one of the most prevalent and intractable clinical symptoms of neuropathic pain. Moreover, the precise pathogenic neuroimmune interfaces for Th cells remain controversial, not to mention the detailed pathogenic mechanisms. In this review, depending on the biology of Th cells in a neuroimmunological perspective, we summarize what is currently known about Th cells as a trigger for chronic tactile allodynia after nerve injuries, with a focus on identifying what inconsistencies are evident. Then, we discuss how an interdisciplinary perspective would improve the understanding of Th cells as a trigger for chronic tactile allodynia after nerve injuries. Finally, we hope that the expected new findings in the near future would translate into new therapeutic strategies via targeting Th cells in the context of precision medicine to either prevent or reverse chronic neuropathic tactile allodynia.

## Background

Neuropathic pain is a debilitating category of pathological pain caused by a heterogeneous repertoire of lesions or diseases of the somatosensory system, which can result in either heightened or disordered transmission of sensory signals into the spinal cord and the brain [1]. The most common conditions associated with neuropathic pain involve injuries to peripheral nervous system (PNS), such as mechanical nerve trauma or compression (painful radiculopathies included), postherpetic neuralgia (PHN), painful diabetic neuropathy (PDN), chemotherapy-induced peripheral neuropathy (CIPN), and trigeminal neuralgia (TGN) [1]. This chronic disease typically manifests as an increased or altered sensitivity to mechanical or thermal stimuli (hyperalgesia or allodynia). It is resistant to conservative medical management and significantly decreases the quality of life [2]. Therefore, the effective therapeutic options to either prevent or reverse chronic neuropathic pain is a critical unmet need for both public health and clinical practice [1].

It is well-recognized that, even with the same nerve injuries, some individuals go on to develop chronic neuropathic pain, while others experience a resolution of acute neuropathic pain [3, 4]. However, the susceptibility/resilience factors and mechanisms for chronic neuropathic pain after nerve injuries are just at the beginning to be elucidated [4, 5]. This is a crucial scientific problem because elucidating why and how individuals develop or withstand chronic neuropathic pain after nerve injuries will pave the way of translational pain medicine for the development of new therapeutic strategies in the context of precision medicine to either prevent or reverse chronic neuropathic pain [5].

Tactile allodynia is one of the most prevalent and intractable clinical symptoms of neuropathic pain after nerve injuries [2, 6]. Mechanistically, tactile allodynia ultimately arises because of the disordered transmission of touch sensory signals, mainly from myelinated low-threshold mechanoreceptors (LTMRs), into the nociceptive circuitry at varying levels of the nervous system, resulting in the erroneous perception of touch as pain [6–8]. Neuronal plasticity has been shown as the fundamental process for the initiation, chronification, and maintenance of neuropathic pain [9]. Interestingly, pain research in the past two decades has established that neuroinflammation is a key driving force for neuronal plasticity underlying neuropathic pain, including tactile allodynia [10, 11]. While many of current studies focus on the onset of neuropathic pain during the acute phase following nerve injuries [5], accumulating evidence indicates that distinct mechanisms engage when neuropathic pain progresses [10]. For instance, after nerve injuries, while microglia are rapidly activated to promote the onset of neuropathic pain [12], astrocytes are activated with a delay of several days or weeks and drive the chronification and maintenance of neuropathic pain rather than its initiation [13]. In this review, we therefore give a brief overview of the key findings regarding the neurobiological and immunological mechanisms for how tactile allodynia gets stuck after nerve injuries in susceptible individuals.

Major histocompatibility complex class II (MHCII)-restricted T helper (Th) cells, a pivotal category of the heterogeneous T cell population [14], have been shown as an important trigger for chronic tactile allodynia after nerve injuries [15]. However, certain limitations for the current state of preclinical and clinical evidences must be faced up to. Moreover, it remains controversial as to where along the neuroaxis Th cells act as a trigger for chronic tactile allodynia after nerve injuries [15]. The uncertainty of the pathogenic neuroimmune interfaces for Th cells presents an inescapable obstacle for further insights into the detailed mechanisms for Th cells as a trigger for chronic tactile allodynia after nerve injuries. The essential reason underlying the current dispirited state is that these neuroimmune studies are, more often than not, designed by and for neuroscientists themselves. The inadequate adoption of immunological perspective, nomenclature, and techniques of Th cells makes the current evidences inconsistent.

With the simple import of immunological nomenclature and techniques, recent studies have rapidly provided strong contrasting evidence to the prevailing notions and have demonstrated the absence of blood-derived monocyte infiltration into the spinal cord dorsal horns (SC-DHs) after nerve injuries [16–20]. Therefore, we focus here on an interdisciplinary perspective, Th cell neuroimmunology in particular, that would benefit neuroscientists who desire deeper insights into pain neuroimmunology. First, we introduce the biology of Th cells in a neuroimmunological perspective. Then, we summarize what is currently known about Th cells in the development of chronic tactile allodynia after nerve injuries, with a focus on identifying what inconsistencies are evident. Finally, we discuss how an interdisciplinary perspective would result in a more comprehensive understanding of the detailed roles and mechanisms of Th cells as a trigger for chronic tactile allodynia after nerve injuries. This knowledge would ultimately herald a new era for either preventing or reversing chronic neuropathic tactile allodynia via targeting Th cells in the context of precision medicine.

### The acute to chronic transition of tactile allodynia after nerve injuries

**Fig. 1:**
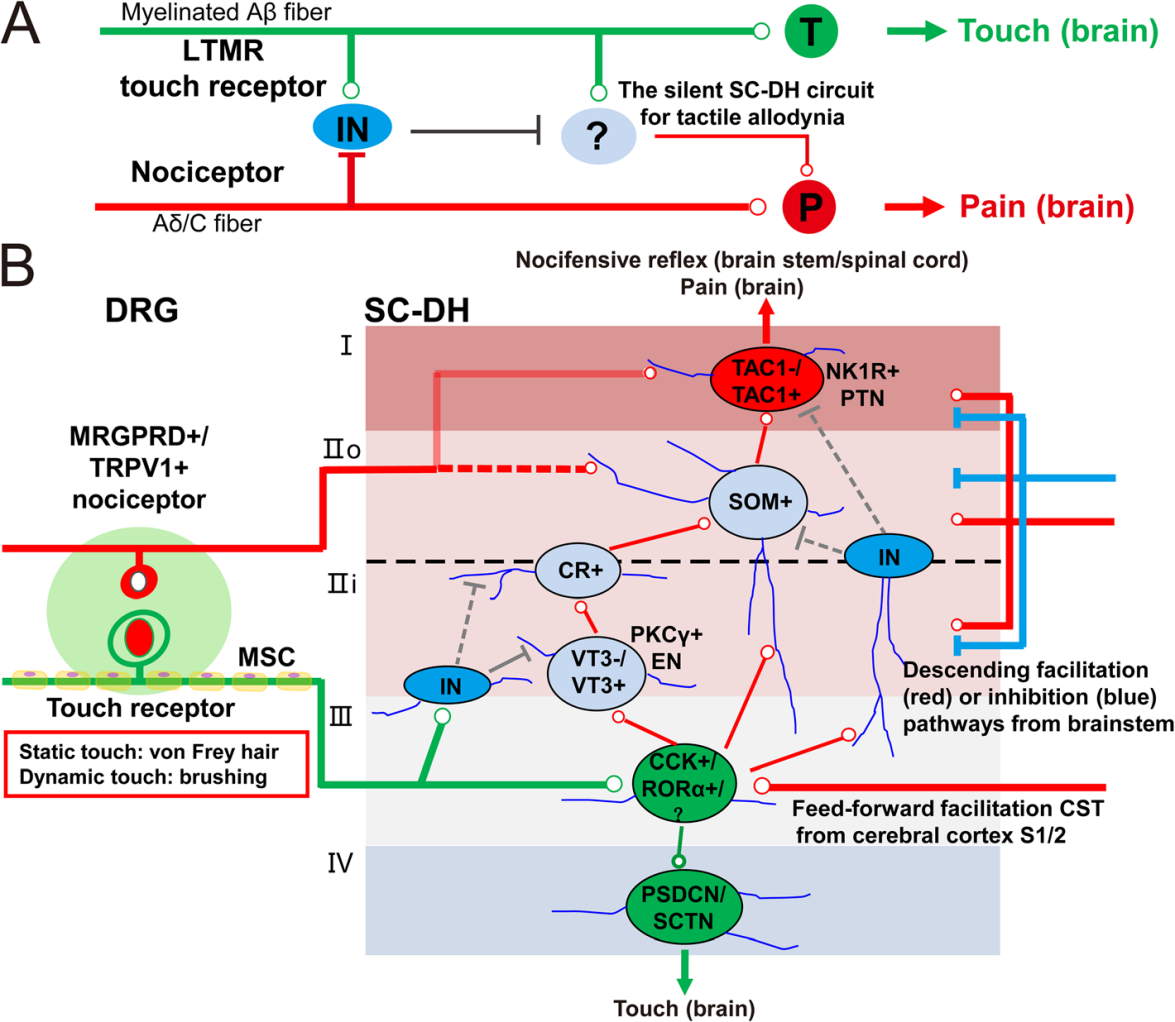
The SC-DH circuit for the transformation of touch into pain (i.e., tactile allodynia) after nerve injuries. **a** Neural circuit framework of neuropathic tactile allodynia based on the gate control theory of pain. **b** Schematic illustration of the SC-DH circuit for tactile allodynia after nerve injuries. While static tactile allodynia evoked by von Frey hairs is dependent on VT3^−^ excitatory interneurons, morphine-resistant, dynamic tactile allodynia evoked by brushing is dependent on VT3^+^ excitatory interneurons. Moreover, while TAC1^−^NK1R^+^ PTNs are sufficient for reflexive defensive reactions, TAC1^+^NK1R^+^ PTNs are necessary for sustained pain-associated coping behaviors. CCK, cholecystokinin; CR, calretinin; CST, corticospinal tract; DRG, dorsal root ganglion; EN, excitatory interneuron; IN, inhibitory interneuron; LTMR, low-threshold mechanoreceptor; MRGPRD, MAS related GPR family member D; MSC, myelinated Schwann cell; NK1R, neurokinin 1 receptor; PKCγ, protein kinase C gamma; PSDCN, postsynaptic dorsal column neuron; PTN, pain transmission neuron; RORα, retinoid receptor-related orphan receptor; SC-DH, spinal cord dorsal horn; SCTN, spino-cervical tract neuron; SOM, somatostatin; TAC1, preprotachykinin 1; TRPV1, transient receptor potential cation channel, subfamily V, member 1; VT3, vesicular glutamate transporter 3

The sense of touch (pressure is not considered in this review) is essential for daily activities throughout our lives, as it provides the vital real-time information about the nature of our physical environment [21]. In the periphery, touch sensations are mainly conveyed by myelinated LTMRs, which are upstream-activated by their innervated mechanosensory end organs. Then, the innocuous touch input from the periphery is transmitted to deep dorsal horn (DH) circuits within the spinal cord (SC) and ultimately relayed to the somatosensory cortex for the perception of touch [21]. At the same time, myelinated LTMR inputs are feedforward gated to prevent the activation of spinal pain transmission neurons (PTNs) within the superficial DH via physiologically silent, dorsally directed neural microcircuits (Fig. 1) [6, 22]. Therefore, touch is not perceived as pain under physiological conditions.

**Fig. 2:**
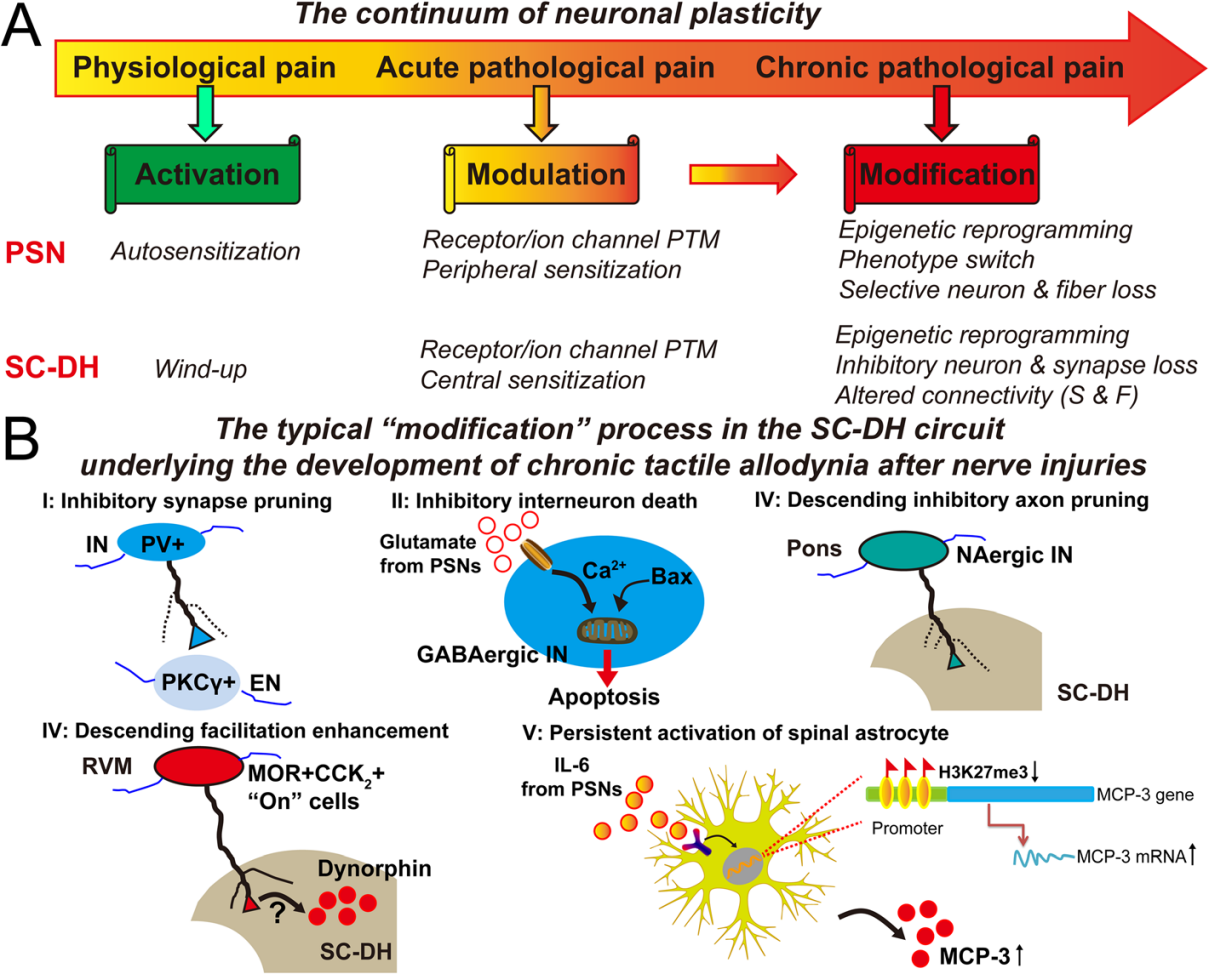
The acute to chronic transition of tactile allodynia after nerve injuries. **a** Schematic illustration of the continuum of neuronal plasticity ranging from “activation” to “modulation” and “modification” for the development of distinct pain states. Detailed forms of neuronal plasticity for PSNs and SC-DH neurons are shown in brief. **b** Schematic summary of the typical “modification” processes in the SC-DH circuit underlying the development of chronic tactile allodynia after nerve injuries. CCK_2_, cholecystokinin type 2 receptor; EN, excitatory interneuron; F, function; IL-6, interleukin-6; IN, inhibitory interneuron; MCP-3, monocyte chemotactic protein-3; MOR, μ opioid receptor; PKCγ, protein kinase C gamma; PSN, primary sensory neuron; PTM, post-translational modification; PV, parvalbumin; RVM, rostral ventromedial medulla; S, structure; SC-DH, spinal cord dorsal horn

Following nerve injuries, the innocuous touch become painful. The erroneous perception of touch as pain involves robust neuronal changes in the key SC-DH circuits responsible for tactile allodynia in the form of “modulation” or “modification” (Fig. 2a) [6, 9]. Aberrant primary afferent input and altered descending supraspinal input collectively drive neuronal plasticity in the SC-DH [23, 24]. The resulting central sensitization (including facilitation and disinhibition) within the SC-DH circuits opens or overcomes the inhibitory gates for LTMR inputs, thus allowing innocuous touch stimuli to directly activate physiologically silent and polysynaptic neural microcircuits linked to pain sensation (Fig. 1) [22–24]. Moreover, during this painful transformation of touch, neuroinflammation across the somatosensory pathway has been shown to play an active role [6, 10]. Detailed information about the current state of evidence for the transformation of touch into pain after nerve injuries has been summarized in recent review articles [6, 25].

However, how tactile allodynia becomes chronic after nerve injuries is one of the most important but still unclear questions at this moment. Therefore, we instead give particular attention to the key findings regarding the neurobiological and immunological mechanisms for the acute to chronic transition of tactile allodynia in susceptible individuals after nerve injuries (Fig. 2b). In essence, transition to chronic tactile allodynia after nerve injuries is the “modification” process establishing stable structural/functional microcircuits for persistent but disordered transmission of touch sensory signals into the nociceptive circuitry in the SC-DH (Fig. 2a) [3, 9].

Local modifications within the SC-DH have been shown to underlie the development of this chronic condition. The most prominent example is persistent local disinhibition, in the form of the robust pruning of inhibitory synapses from parvalbumin (PV) interneurons onto PKCγ excitatory interneurons [26] or the excitotoxic cell death of γ-aminobutyric acid (GABAergic) inhibitory interneurons [27]. Modifications in descending supraspinal inputs are also engaged. On the one hand, nerve injuries can result in significant loss of descending pontospinal noradrenergic inhibitory fibers within the SC-DH [28]. On the other hand, nerve injuries lead to delayed enhancement of descending facilitation from “On” cells in the rostral ventromedial medulla (RVM), which co-express mu opioid receptor (MOR) and cholecystokinin type 2 receptor (CCK_2_) [29]. This results in persistent increase of spinal dynorphin, which maintains neuropathic pain [30, 31].

For the involvement of neuroinflammation, the most prominent example is the delayed but persistent activation of astrocytes within the SC-DH in response to nerve injuries [32]. Nerve injury induces interleukin 6-dependent histone demethylation at the promoter of monocyte chemotactic protein 3 (MCP-3) gene mostly in spinal astrocytes. This epigenetic modification results in persistent increase of MCP-3 expression and secretion in activated spinal astrocytes. MCP-3 further acts as a critical driver for either astrocyte-microglia interaction or neuronal sensitization within the SC-DH to maintain neuropathic pain, including tactile allodynia [33].

## The biology of Th cells: a neuroimmunological perspective

### What are Th cells?

**Fig. 3:**
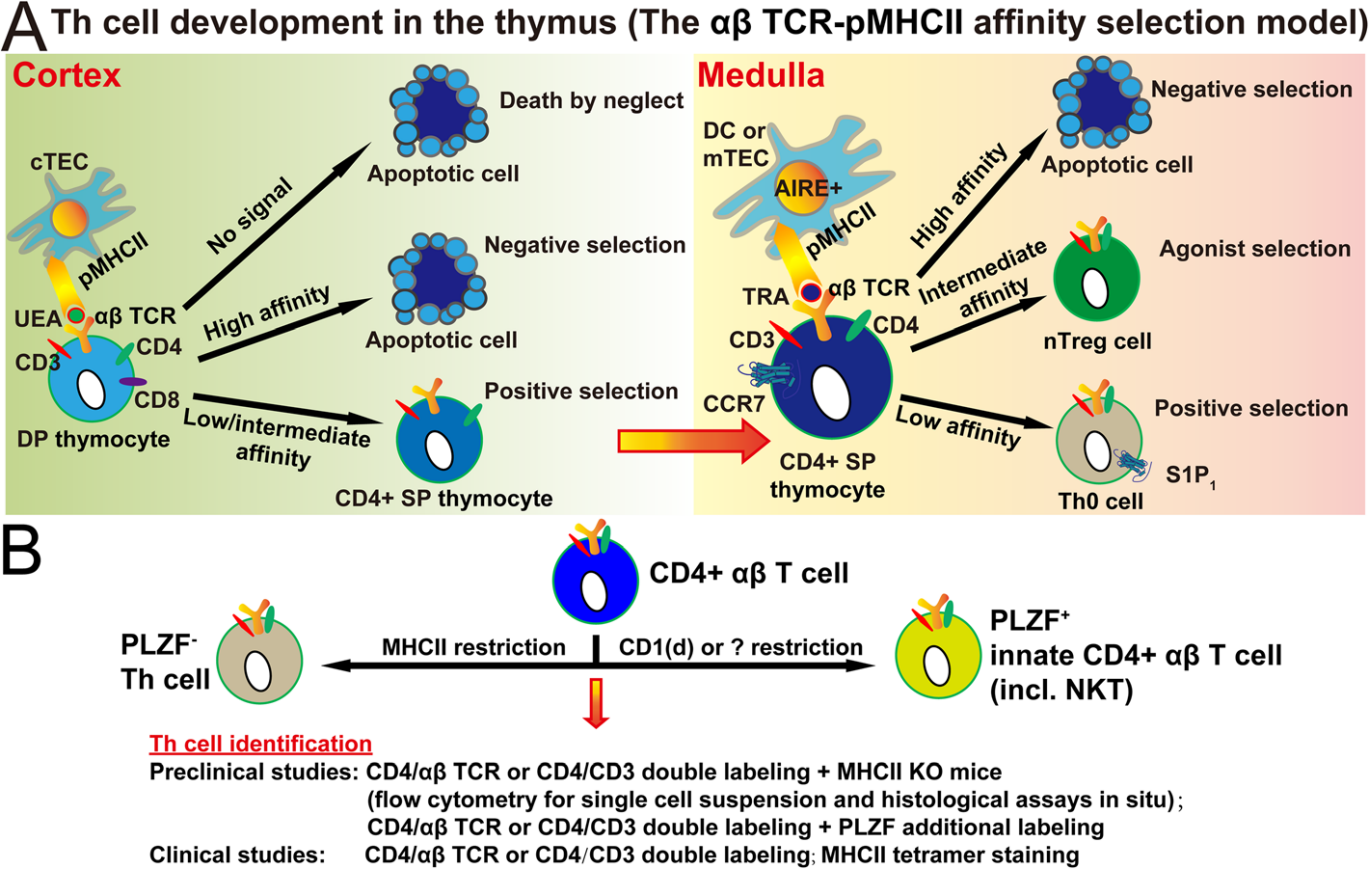
The definition and identification for Th cells from the perspective of thymic development of Th cells. **a** Schematic summary of the development of Th cells in the thymus cortex and medulla. **b** Schematic illustration of Th cells versus innate T cells among CD4^+^ αβ T cells. Th cells are, in the strict definition, MHCII-restricted CD4^+^ αβ T cells and PLZF negative. The identification methods for Th cells are shown. DC, dendritic cell; PLZF, promyelocytic leukemia zinc finger; TEC, thymic epithelial cell; TRA, tissue-restricted antigen; UEA, ubiquitous-expressed antigen

It has been a consensus that Th cells is the central player in orchestrating innate and adaptive immune responses, depending on αβ T cell receptor (TCR) recognition of cognate peptide-MHCII (pMHCII) complex [14, 34]. Th cells are developed in the thymus (Fig. 3a) [35, 36]. In brief, the developing CD4^+^CD8^+^ double-positive (DP) thymocytes are first instructed in the thymic cortex to differentiate into CD4^+^CD8^−^ single-positive (SP) thymocytes via the interactions with cortical thymic epithelial cells (cTECs). After chemotaxis into the thymic medulla, SP thymocytes are further instructed for the differentiation into naïve Th cells via the interaction with either medullar thymic epithelial cells (mTECs) or migratory dendritic cells (DCs). Both thymic cortical and medullar instruction relies on the affinity/avidity with which αβ TCRs of the developing thymocytes bind endogenous pMHCII complexes on thymic antigen-presenting cells (APCs). This thymic selection lays down the first checkpoint to ensure that the peripheral Th cell repertoire is capable of mounting effective immune responses against foreign antigens, while minimizing the risk of autoimmunity to ubiquitous-expressed antigens (UEAs) and tissue-restricted antigens (TRAs) [37].

Hence, Th cells are, in the strict definition, MHCII-restricted CD4^+^ αβ T cells (Fig. 3b). Since there are few, if any, CD4^+^ γδ T cells [38], Th cells are conventionally named as CD4^+^ T cells and identified as CD3^+^ CD4^+^ cells by histology in situ or flow cytometry in single-cell suspension (Fig. 3b). However, CD4^+^ αβ T cells or CD4^+^ T cells are composed of not only a major population of MHCII-restricted Th cells, but also a minor population of innate CD4^+^ αβ T cells, including CD1d-restricted natural killer T cells (NKTs) (Fig. 3b) [39]. There are currently no unique molecular markers to directly distinguish Th cells from innate CD4^+^ αβ T cells among CD4^+^ αβ T cells or CD4^+^ T cells. The main reason for this technical challenge is that Th cells and innate CD4^+^ αβ T cells often express a common or similar set of transcriptional factors, cytokines, and surface molecules [40]. Therefore, the most accurate identification of Th cells requires combinatorial use of CD4/αβ TCR or CD4/CD3 double labeling as Th cell detection method and MHCII knockout animals as Th cell depletion method (Fig. 3b). However, this identification strategy is achievable only in preclinical studies, especially in mice [41], rather than clinical studies. In the case that the cognate antigens are known, MHCII tetramer staining is the ideal methods to identify Th cells in clinical studies (Fig. 3b) [42].

The promyelocytic leukemia zinc finger (PLZF) is a specific key transcription factor for the development of innate CD4^+^ αβ T cells but not for Th cells [43–46]. It has been reported that PLZF expression is highly specific to innate T cells, rather than conventional T cells (including Th cells). Furthermore, PLZF expression cannot be induced de novo in conventional T cells via TCR-mediated activation or inflammation [47]. Therefore, PLZF could be used as a potential effective molecular biomarker to directly distinguish PLZF^−^ Th cells from PLZF^+^ innate CD4^+^ αβ T cells for technical practice (Fig. 3b). However, this potential molecular biomarker requires further validation of the ubiquitous expression of PLZF in all the innate CD4^+^ αβ T cells.

### How do Th cells function?

**Fig. 4:**
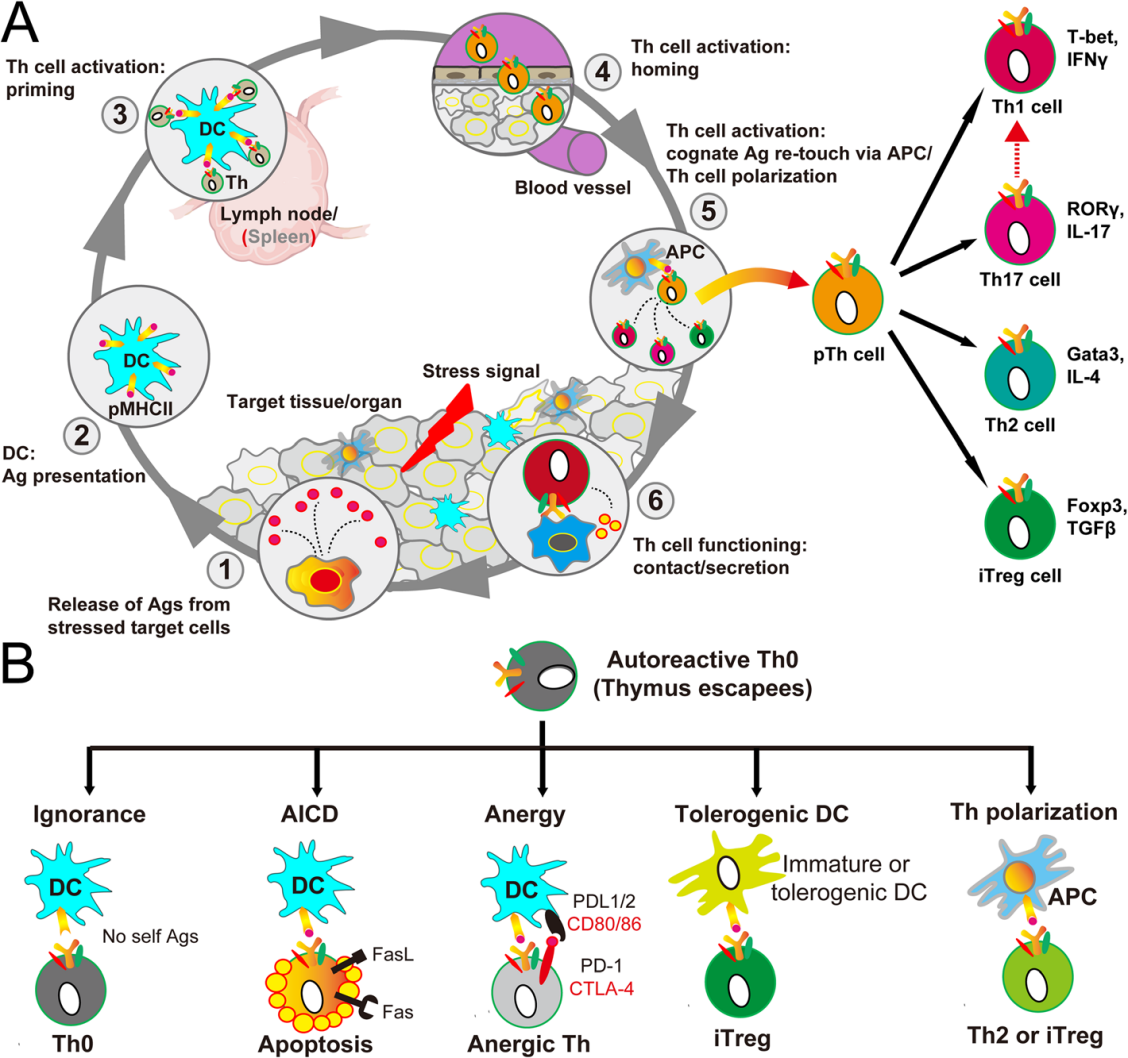
The biology of Th cells at the periphery. **a** Schematic illustration of the activation of Th cells upon antigen stimulation at the periphery. According to “the second touch” hypothesis, full Th polarization needs a second antigen presentation to primed Th cells by APCs in the inflamed tissues. **b** Schematic summary of peripheral tolerance mechanisms against autoreactive Th cells, which escape from thymic negative selection. Ag, antigen; AICD, activation-induced cell death; APC, antigen-presenting cell; DC, dendritic cell; pTh, primed Th cell

Following maturation in the thymus, naïve Th cells enter into the blood circulation and traffic between secondary lymphoid organs (the spleen and lymph nodes) and the blood. Upon antigen stimulation, naïve Th cells are confronted with three integrated tasks to fulfill their critical roles in immunity (Fig. 4a). First, naïve Th cells must decide whether to turn on or off an immune response. Second, if an immune response is turned on, the proper effector classes of Th cells must be decided before functioning via the manner of contact/secretion in the inflamed target tissues. Third, memory Th cells, either effector or central, should be formed to remember past encounters.

In secondary lymphoid organs, through dynamic contact with DCs in the form of immunological synapses, naïve Th cells are activated and expanded by two signals from the same DC: one from αβ TCR/CD4 engagement with antigenic peptide in the context of MHCII and the other one through CD28 engagement by CD80 and/or CD86 (Fig. 4a) [34, 48]. Activated Th cells are checked for survival and then acquire an array of activation markers [34].

Upon activation, the proper effector classes of Th cells must be decided to fulfill their essential role in immune responses (Fig. 4a). The main subsets of Th cells are Th1, Th2, Th17, inducible Treg (iTreg), follicular T helper (TFH), and follicular Treg (fTreg) cells [14, 34]. Traditional view holds that activated Th cells are fully polarized to particular Th subsets directly in secondary lymphoid organs. The cytokine milieu that is generated by APCs is an important factor that influences the differentiation of activated Th cells [14, 34, 48]. However, antigen-experienced Th cells acquire peripheral homing receptors soon after their activation [49]. This forces these primed Th cells to rapidly return to the tissue where the antigen resides, which would result in incomplete polarization of activated Th cells in secondary lymphoid organs per se [50]. Accumulating evidence indicated that additional antigen presentation to primed Th cells takes place in the non-lymphoid tissues where the antigen resides [51, 52]. This led to the proposal of “the second touch hypothesis,” which argued that full Th cell polarization into the proper effector classes requires a second antigen presentation to primed Th cells by APCs, either DCs or macrophages, in the inflamed tissues [34].

Autoreactive Th cells, which escape from thymic negative selection, are further checked robustly at the periphery to prevent the development of autoimmunity (Fig. 4b) [37]. First, autoreactive Th cells can ignore self-antigens that are not adequately presented by DCs. The inadequate presentation may result from the low accessible amount of self-antigens per se or the biased peptide presentation of a particular MHCII haplotype. Then, if self-antigens are adequately presented by DCs, autoreactive Th cells can be checked via tuning the fates of activation. Although mature DCs sample and present self-antigens to autoreactive Th cells, these Th cells may either depleted via activation-induced cell death (AICD) or inactivated to become unresponsive or anergic Th cells. Immature or tolerogenic DCs also continuously sample and present self-antigens to autoreactive Th cells, but bias Th cell differentiation into iTreg cells to suppress autoimmunity. Last but not the least, after their activation by mature DCs, autoreactive Th cells can be checked at the point of polarization to avoid autoimmunity. Low or intermediate TCR signal intensity relative to the threshold of Th cell activation has been reported as a determinant for iTreg/Th2 cell polarization to suppress autoimmunity [53, 54].

### How to methodologically define Th cell effector subsets?

At present, phenotypic characterization of Th cell effector subsets is defined by the signature cytokines or chemokine receptors that the Th cell subset secretes or expresses and by the master transcription factors upon which the Th cell subset arises [14, 34]. In detail, among Th cells, Th1 cells are identified as T-bet^+^IFNγ^+^ cells, Th2 cells as Gata3^+^IL-4^+^ cells, Th17 cells as RORγ^+^IL-17^+^ cells, iTreg cells as Foxp3^+^TGFβ^+^ cells, TFH cells as Bcl-6^+^CXCR5^+^ cells, and fTreg cells as Foxp3^+^CXCR5^+^ cells (Fig. 4a). Over the years, flow cytometry for immune cell suspensions and histological assays in situ with selected molecular markers have established themselves for phenotypic characterization of Th cell subsets [55].

However, additional complexity in the differentiation of Th cells is becoming evident. On the one hand, it remains controversial whether each subset of Th cells is firmly fixed or remains plastic [14]. For instance, under some circumstances, Th17 cells can also produce IFNγ, the signature cytokine of Th1 cells [14, 56]. More importantly, the effector functions of Th17 cells in autoimmune diseases require their transdifferentiation into Th1-like cells via metabolic reprogramming [56]. The co-expression of Foxp3 and RORγt, the master transcription factor for iTreg and Th17 cells, respectively, has been seen in the same Th cell. Consequently, it seems that some iTreg cells can be induced to transdifferentiate into Th17 cells [14]. On the other hand, accumulating evidence suggests diverse functional states of a particular subset of Th cells. For instance, in vitro polarized Th17 cells can either cause severe autoimmune responses upon adoptive transfer, or have little or no effect in inducing autoimmune disease [57]. Therefore, the complexity of Th cell polarization renders doubtful the use of the selected but limited molecular markers to define a particular subset of Th cells [55].

### How do Th cells enter the CNS?

**Fig. 5:**
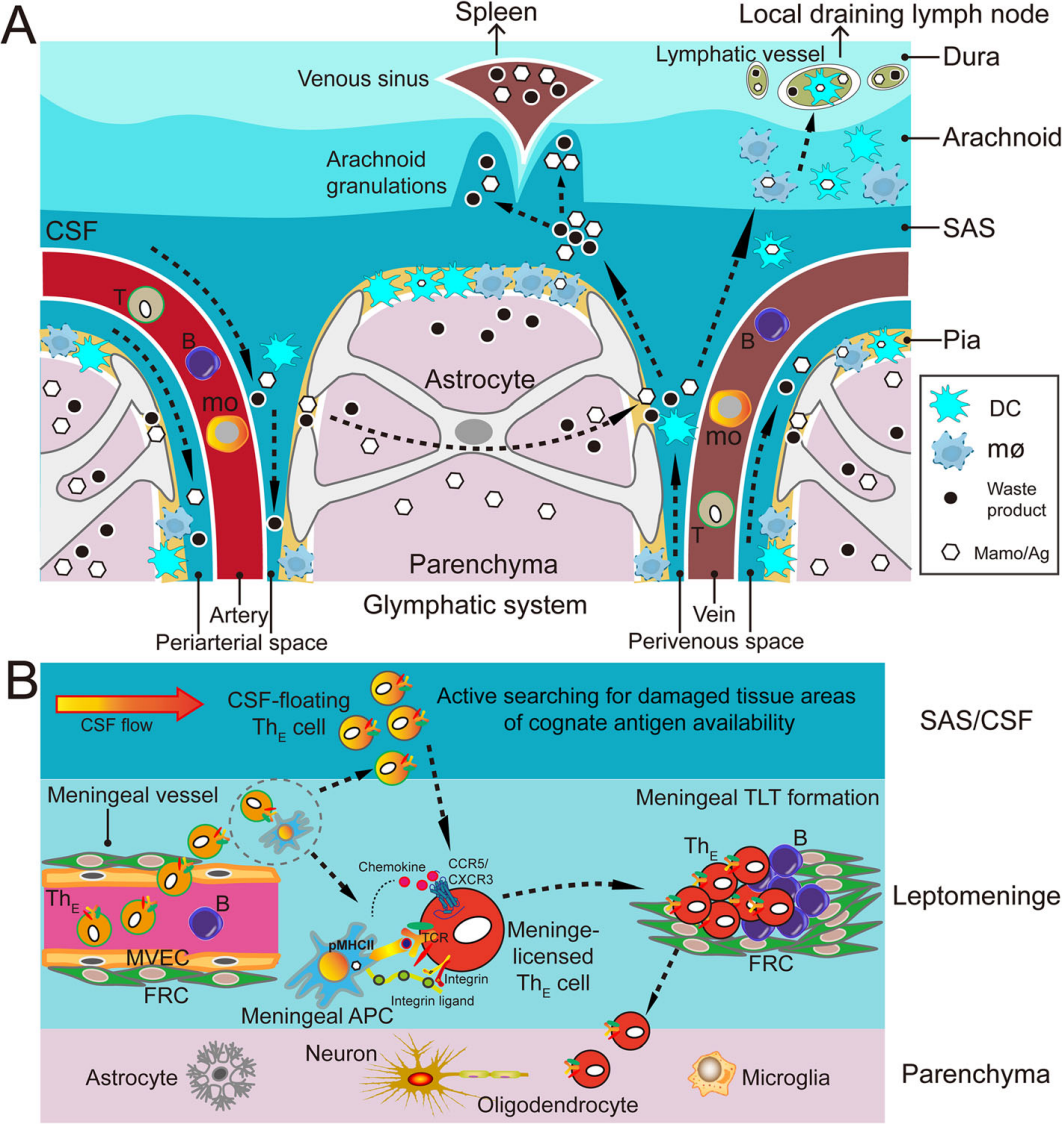
The cerebrospinal meninges in the pathogenesis of experimental autoimmune encephalomyelitis, a Th cell-mediated autoimmune disease. **a** Schematic illustration of the anatomy and histology of cerebrospinal meninges from the neuroimmunological point of view. The communication of the glymphatic system and the cerebrospinal meninges is shown. **b** The infiltration of pathogenic Th cells into the cerebrospinal meninges before their infiltration into the CNS parenchyma. Ag, antigen; APC, antigen-presenting cell; CSF, cerebrospinal fluid; DC, dendritic cell; FRC, fibroblastic reticular cell; mamo, macromolecules; mo, monocyte; mø, macrophage; MVEC, meningeal vascular endothelial cell; SAS, subarachnoid space; Th_E_, effector Th cell; TLT, tertiary lymphoid tissue

As stated in the “Preclinical evidences” section, the effector immune functions of Th cells, excepting TFH and fTreg cells, require the homing of primed Th cells back to the inflamed target tissues where the corresponding antigen resides. During immune responses in the central nervous system (CNS), traditional views hold that Th cells infiltrate the inflamed nervous tissues via the dysfunctional blood-brain barrier (BBB). However, a growing body of evidence indicates that the infiltration of pathogenic Th cells into the CNS parenchyma is likely secondary to their infiltration into the cerebrospinal meninges during neuroinflammation [58]. Over the past decades, accumulating anatomical and histological studies of the cerebrospinal meninges provide the structural basis for the cerebrospinal meninges as a critical neuroimmune interface for both beneficial and detrimental cross-talks between adaptive immunity and the CNS during homeostasis and diseases (Fig. 5a) [58–60].

For instance, in rat experimental autoimmune encephalomyelitis (EAE), a model of multiple sclerosis, the spinal leptomeninges are shown as a checkpoint where activated Th cells are licensed to enter the CNS parenchyma. Here, circulating effector Th cells have been shown to get arrested to leptomeningeal vessels and immediately monitor the luminal surface, crawling preferentially against the blood flow (Fig. 5b) [61]. After diapedesis, effector Th cells are anchored in the 3D leptomeningeal network of collagen fibers for effective licensing via leptomeningeal macrophages which effectively present cognate antigens derived from myelin proteins. This meningeal licensing is achieved via antigen-specific physical contacts with resident macrophages, chemokine signaling via CCR5/CXCR3, and integrin signaling (Fig. 5b) [52]. The non-licensed Th cells are preferentially released into the cerebrospinal fluid (CSF), from where they can actively search for damaged nervous tissue areas of cognate antigen availability. Upon arriving at the leptomeninges around these tissue areas, Th cells are licensed there via the same mechanisms mentioned above (Fig. 5b) [52]. Moreover, with the co-operation of meningeal stromal cells, such as fibroblastic reticular cells (FRCs), effector Th cells induce the formation of tertiary lymphoid tissues (TLTs) in the cerebrospinal meninges. The de novo TLTs enable further effector Th cells to reside within the meninges (Fig. 5b) [60].

## Emerging roles of Th cells in the transition to chronic tactile allodynia after nerve injuries

In this section, we summarize both clinical and preclinical evidences showing Th cells, the orchestrator of an immune response, as an emerging trigger for chronic tactile allodynia after nerve injuries. Notably, we focus here on primary nerve injuries per se, rather than nerve injuries secondary to autoimmune attacks or infections, such as Guillain-Barre syndrome (GBS) and PHN. In these primarily immune-drived nerve injuries, it is technically difficult to ascertain specific roles of Th cells in the pathogenesis of primary nerve damage versus secondary neuropathic pain.

### Clinical evidences

**Fig. 6:**
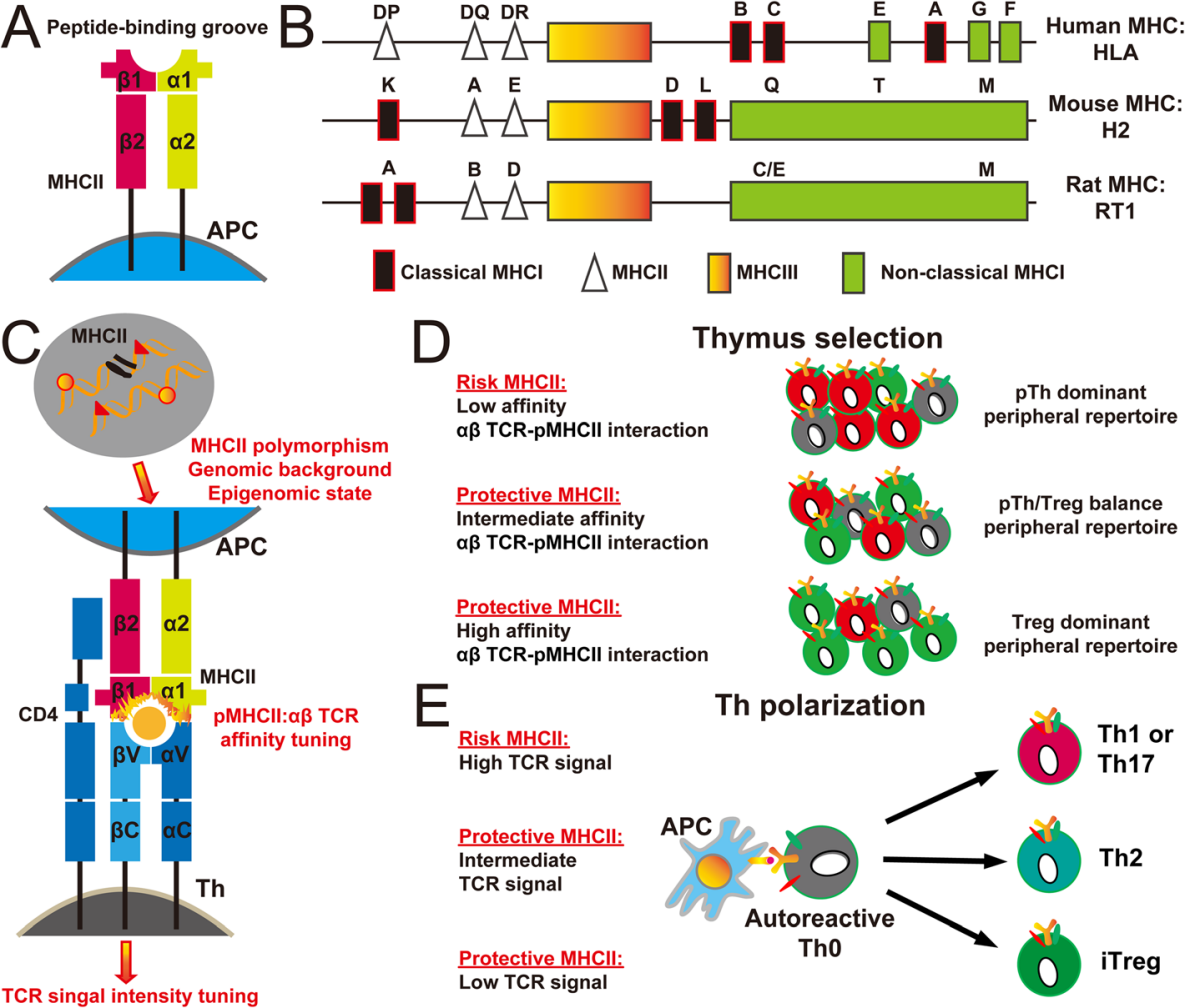
MHCII gene polymorphism and Th cell-mediated autoimmunity. **a**, **b** Schematic illustration of molecular structure of MHCII (**a**) and genetic structure of MHCII (**b**). **c** On particular genomic backgrounds and epigenomic states, MHCII gene polymorphism fine tunes the affinity of pMHCII: TCR interactions and the TCR signal intensity. **d**, **e** The role of MHCII gene polymorphism in the susceptibility or resistance of Th cell-mediated autoimmunity through modulating thymus selection and Th polarization. APC, antigen-presenting cell; HLA, human leukocyte antigen; MHC, major histocompatibility complex; pTh, pathogenic Th cells for autoimmunity; TCR, T cell receptor

MHCII (Fig. 6a, b) is specifically required for thymic development and peripheral activation of Th cells (Figs. 3a and 4a). Furthermore, MHCII gene polymorphism has been implicated in establishing or breaking central and peripheral tolerance for Th cells via fine tuning the affinity of pMHCII: TCR interactions and the TCR signal intensity (Fig. 6c, d, e) [62–68]. Therefore, MHCII gene polymorphism is associated with the susceptibility or resistance to Th cell-mediated autoimmunity. In humans, major MHCII isoforms for antigen presentation to Th cells by APCs are from the families of human leukocyte antigen (HLA)-DP, DQ, and DR (Fig. 6b). Recent clinical studies have shown an association of human MHCII gene polymorphism with the susceptibility to chronic neuropathic pain after nerve injuries. In particular, the DQB1*03:02 HLA haplotypes has been shown to have an increased risk for the development of chronic neuropathic pain after inguinal hernia surgery and lumbar disc herniation (LDH) [69]. Likewise, human whole blood transcriptomic profiles showed that the significant upregulation of MHCII antigen presentation pathway genes is associated with the vulnerability of the transition from acute to chronic low back pain, a prevalent form of neuropathic pain [70]. Therefore, these clinical evidences indicate that Th cells may play an initiating role in the development of chronic neuropathic pain, including chronic tactile allodynia.

In support of this suggestion, a growing body of clinical evidences has shown the changes of Th cell numbers and subset patterns in peripheral blood specifically in patients with chronic neuropathic pain after nerve injuries. First, compared with health controls, the counts of CD4^+^ T cells (presumably Th cells) in patients with LDH were shown to be increased and positively correlated with neuropathic pain intensities [71]. In particular, the numbers of Th17 cells were further shown to be higher in patients with ruptured than non-ruptured lumbar discs and also correlated with neuropathic pain intensities [72]. Second, both central and effector memory CD4^+^ T cells were shown to be significantly increased in chronic neuropathic pain patients with carpal tunnel syndrome (CTS) and complex regional pain syndrome (CRPS), as compared to healthy controls. The upregulation of memory CD4^+^ T cells (presumably Th cells) would be an indicator of the history of Th cell activation against a particular antigenic stimulus in CTS patients [73, 74]. To our surprise, however, significant decrease of inflammatory Th1 or Th17 cells and increase of anti-inflammatory Th2 or Treg cells were found in patients with chronic neuropathic pain as compared to healthy controls [75–77].

### Preclinical evidences

In consistent with clinical studies, preclinical studies further support Th cells as an emerging trigger for chronic tactile allodynia after nerve injuries. As in clinical studies, the MHC gene polymorphism has been seen to influence the susceptibility to this chronic condition after nerve injuries in rats, although whether the major MHC haplotype-specific effect on disease susceptibility is mapped to the MHCII gene region need to be determined in detail [78]. Moreover, during the sub-acute phase after nerve injuries in mice or rats, CD4^+^ T cells (presumably Th cells), rather than other immune cells (such as CD8^+^ T cells, B cells, natural killer (NK) cells, and macrophages), were found to be selectively activated by DCs and polarized to IFNγ-positive, inflammatory Th1 cells in peripheral lymphoid organs, such as the spleen and local draining lymph nodes [15, 79–81]. The distinct involvement of Th subsets after traumatic nerve injuries in clinical and preclinical studies may due to the plasticity of Th cells, especially Th17 cells (Fig. 4a) [56].

In addition, both CD45^+^CD4^+^ lymphocytes (possibly Th cells) and CD45^+^CD8^+^ lymphocytes were found to be significantly increased in the blood 7 but not 13 days after high-dose paclitaxel induction in adult male C57BL/6 J mice [82]. However, there were no significant changes for CD3-positive T cells in the blood 7 days after low-dose paclitaxel induction in adult male C57BL/6 J mice [83]. Moreover, for peripheral lymphoid organs, such as the spleen and lymph nodes, CD45^+^CD4^+^ lymphocytes and their Th1/Th17/Th2/Treg subsets were not found to be significant changed 7 or 13 days after high-dose paclitaxel and oxaliplatin induction in adult male C57BL/6 J mice, except for transient increase in splenic Th2-like cells and sustained increase in nodal Treg cells for oxaliplatin-treated mice [82]. The discrepancy may arise from the detection timepoints relative to CIPN models and sample locations. These indirect preclinical evidences suggest that Th cells may contribute to the transition to chronic tactile allodynia after nerve injuries.

Accumulating studies targeting Th cells have provided more direct preclinical evidences showing Th cells as an emerging trigger for chronic tactile allodynia after nerve injuries (Table 1). First, studies using genetically immunodeficient mice (including RAG1/2 knockout mice, SCID mice, nude mice or rats, and B cell-deficient mice) and T cell reconstitution have demonstrated that T cells, but not B cells, are essential for the development of chronic tactile allodynia after nerve injuries [79, 80, 84–87]. Furthermore, passive transfer of nerve injury-conditioned, type 1 polarized T cells (presumably Th1 cells) from heterozygous rats into athymic nude rats restored the level of tactile allodynia in the recipients to that of heterozygous donor rats [80]. Therefore, Th cells, and even Th1 cells, may contribute to the development of chronic tactile allodynia after nerve injuries. Second, the transition from acute to chronic tactile allodynia after nerve injuries was shown to be impaired in CD4 knockout mice, which are specifically devoid of CD4^+^ cells, but restored in CD4 knockout mice reconstituted with CD4+ cells [79]. This implies that Th cells may contribute to the development of chronic tactile allodynia after nerve injuries, since the majority of CD4^+^ cells (~ 75%) are CD4^+^ T cells (presumably Th cells) [79]. It is worth noting that CD8^+^ but not CD4^+^ T cell reconstitution in RAG1 knockout mice restored the resolution of acute tactile allodynia which was seen in wild-type mice after paclitaxel or cisplatin induction in adult C57BL/6 male and female mice [83, 88]. Similarly, pharmacological or genetic inhibition of cathepsin S, for which the most important biological function lies in the MHCII antigen presentation pathway and the development and activation of Th cells, significantly attenuated the transition to chronic tactile allodynia after nerve injuries [81]. Furthermore, bolus-adoptive transfer of nerve injury-conditioned splenocytes or splenic CD4+ T cells from wild-type mice into cathepsin S knockout mice or splenectomized mice temporarily restored the level of tactile allodynia in the recipients to that of the donors [81]. Therefore, Th cells were suggested to contribute to the development of chronic tactile allodynia after nerve injuries. Last but the most important, the most direct preclinical evidence has been provided by MHCII knockout mice (specifically devoid of MHCII-restricted Th cells) and show the impaired transition of acute tactile allodynia to a chronic state after nerve injuries [89].

**Table 1:** Chronic tactile allodynia after nerve injuries in rodents with potential Th cell targeting compared to controls

Nerve injuries	Potential techniques for Th cell targeting	Animal species/sexes	Pain severity vs. controls	References
SNI	Rag1^−/−^	C57BL/6 J mice; M	Reduced	Cobos et al. (2018)
	Rag1^−/−^ mice reconstituted with WT T cells		Restored	
SNI	Rag2^−/−^	C57BL/6 mice; M + F	Reduced	Vicuna et al. (2015)
	Rag2^−/−^ mice reconstituted with WT T cells		Restored	
CIPN (Paclitaxel)	Rag1^−/−^	C57BL/6 J mice; M + F	Prolonged	Krukowski et al. (2016)
	Rag1^−/−^ mice reconstituted with CD4^+^ T cells		Unchanged	
CCI	Nude	Lewis rat; M	Reduced	Moalem et al. (2004)
	Transfer of conditioned Th1-like cells from heterozygous rats into athymic nude rats		Restored	
SSNL	CD4^−/−^	BALB/c mice; M	Reduced	Cao et al. (2008)
	CD4^−/−^ mice reconstituted with CD4^+^ cells		Restored	
SSNL	CatS inhibitor Z-FL (i.p.)	DBA/2 mice; M	Reduced	Zhang et al. (2014)
	CatS^−/−^; splenectomy		Reduced	
	Transfer of conditioned splenocytes or splenic CD4+ T cells from WT mice into CatS^−/−^ or splenectomized mice		Restored	
SSNL	MHCII^−/−^	C57BL Mice; M	Reduced	Sweitzer et al. (2002)

*CCI* chronic constriction injury, *CINP* chemotherapy-induced peripheral neuropathy, *F* female, *M* male, *SNI* spared nerve injury, *SSNL* selective spinal nerve ligation

### Limitations to clinical and preclinical evidences

Both clinical and preclinical evidences clearly showed that Th cells are an emerging trigger for chronic tactile allodynia after nerve injuries. However, there are several notable limitations to the current state of evidences. We list the most prominent limitations in the following text.

First, the current clinical studies are not rationally designed. They are lack of independent cohorts for prospective studies to validate the results from the retrospective discovery cohorts [69]. For analyzing Th cell events during the sub-acute phase after nerve injuries, the appropriate biomarkers at the corresponding timepoints might have not be carefully selected in these clinical studies. These limitations make the interpretation of the results from these clinical studies very difficult. For example, it remains to be clarified whether the paradoxical Th1/Th17/Treg imbalance seen in patients with chronic neuropathic pain [75–77] represents an underlying pathophysiological mechanism or just an epiphenomenon as a result of chronic pain-associated, chronic stress [90].

Second, in preclinical studies, accurate targeting and identification of Th cells is not always achieved. Up to now, only one preclinical study used MHCII knockout mice to specifically deplete Th cells to determine their role in the pathogenesis of tactile allodynia after nerve injuries [89]. Moreover, the assessment of tactile allodynia in current preclinical studies solely relies on the paw withdrawal response in the von Frey hair (VFH) test, which has been recognized as a surrogate of static tactile allodynia. However, dynamic tactile allodynia evoked by brushing stimuli is the more clinically relevant form of tactile allodynia, and the role of Th cells in the development of chronic dynamic tactile allodynia has not been determined so far [23]. Moreover, beyond behavioral tests using the paw withdrawal response, additional tests, such as conditioned place aversion (CPA), have been recognized as a necessity for the full assessment of the complex experience of tactile allodynia [91].

Third, there are some common limitations to both preclinical and clinical studies. T cells have been shown to be involved in the development of tactile allodynia, rather than cold allodynia after nerve injuries in male mice [84]. Therefore, future studies are needed to determine the sensory modality specificity for Th cells as a trigger for chronic tactile allodynia after nerve injuries. More importantly, microglia and Th cells have been suggested to be differently engaged in the development of tactile allodynia after nerve injuries in male versus female mice [92, 93]. However, multiple independent studies imply the involvement of Th cells in the transition to chronic tactile allodynia after nerve injuries in male animals (Table 1). Therefore, it remains in both preclinical and clinical studies to further characterize the complex sexual dimorphism for the role of Th cells in the transition to chronic tactile allodynia after nerve injuries. Another limitations that should be overcome is to ascertain whether the role of Th cells in the transition to chronic tactile allodynia after nerve injuries is independent of the skin phenotypes (glabrous versus hairy) and the properties of nerve injuries, such as the type of involved nerves (spinal versus cranial) and damages (mechanical versus non-mechanical).

## The pathogenic neuroimmune interfaces for Th cells as a trigger for chronic tactile allodynia after nerve injuries

**Fig. 7:**
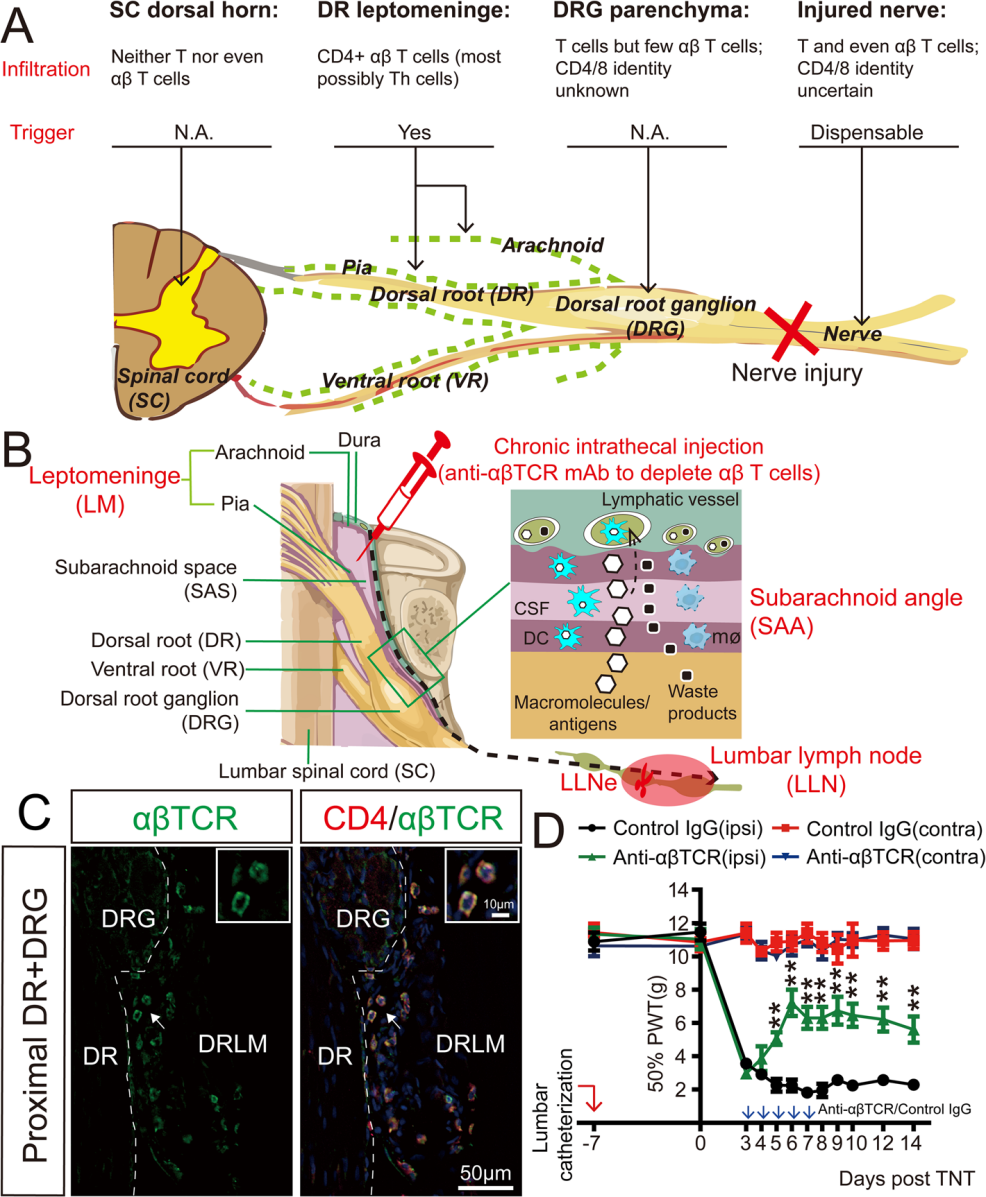
The dorsal root leptomeninges (DRLMs) as the potential neuroimmune interface for Th cells as a trigger for chronic tactile allodynia after nerve injuries. **a** Schematic summary of current evidences for the infiltration of CD4^+^ αβ T cells (most possibly Th cells) along the neuroaxis and functional implications of potential Th cell infiltration in the chronification of tactile allodynia after nerve injuries. **b** Schematic illustration of the anatomy and histology of DRLMs from the neuroimmunological point of view. Region-specific targeting methods for DRLM CD4^+^ αβ T cells (most possibly Th cells) are shown: [1] LLNe: *prior* lymphadenectomy to lumbar lymph nodes (LLNs), where CD4^+^ αβ T cells in DRLMs are derived [2]; chronic intrathecal injection of the suppressive anti-αβTCR antibodies to specifically deplete CD4^+^ αβ T cells that selectively infiltrate into DRLMs along the neuroaxis after nerve injuries. **c** The selective infiltration of CD4^+^ αβ T cells (most possibly Th cells) into lumbar DRLMs along the neuroaxis after adult rat tibial nerve transection (TNT). Here, only the data about DRLMs around the DRG and proximal DR 7 days after nerve injuries is shown for the sake of clarity. **d** Chronic intrathecal application of the suppressive anti-αβTCR antibodies, which specifically deplete CD4^+^ αβ T cells (most possibly Th cells) in DRLMs, reduces the development of chronic tactile allodynia after adult rat TNT

In this section, depending on the perspective of the neuroimmunology of Th cells, especially the nomenclatures and techniques, we summarized what is currently known about the pathogenic neuroimmune interfaces for Th cells in the development of chronic tactile allodynia after nerve injuries, with a focus on identifying what inconsistencies are evident (Fig. 7a).

### Classical pathogenic neuroimmune interfaces

The gray matter of SC-DHs have been classically viewed as an important neuroimmune interface for T cells, including αβ T cells, to initiate the transition to chronic tactile allodynia after nerve injuries [79, 81, 85, 92–97]. However, a growing body of evidence doubts about the presence of αβ T cells, or even T cells there. First, a recent study reported little, if any, CD2-positive cells (presumably T cells) in the SC-DHs over 6 weeks after spared nerve injury (SNI) in adult rats [98], with the same experimental settings as in the seminal study concerning the roles of T cell infiltration into the SC-DHs in nerve injury-induced chronic tactile allodynia [85]. Moreover, another recent study reported little, if any, CD3-positive T cells in the SC-DHs 8 and 28 days after SNI in adult C57BL/6 male and female mice [99]. In our previous study, we also did not find any convincing evidences for the presence of αβ T cells in the SC-DHs 1 weeks after tibial nerve transection (TNT) in adult male *Sprague-Dawley* (SD) rats [15]. Second, after L5 selective spinal nerve ligation (SSNL) in adult male BALB/C or DBA/2 mice, a small number of CD3-positive T cells were found to significantly infiltrate into the SC-DHs [79, 81]. However, there were no T cells detected in the SC-DHs of adult male C57BL/6 mice with the same nerve injury [81]. Third, while a very small number of T cells were found to significantly enter into the SC-DHs after partial sciatic nerve ligation (PSNL) in adult male SD or Wistar rats [94, 97], there were minimal or no T cell (CD3 positive) infiltration into the SC-DHs after PSNL in adult male or female C57BL/6 mice [20, 100, 101]. Fourth, after chronic constriction injury (CCI) to the sciatic nerves of adult male rats, very low densities of αβ T cells relative to the volume of the SC-DHs were detected in the ipsilateral SC-DHs in statistical sense [96, 102–104]. Finally, no CD3-positive T cells were found to significantly infiltrate into the SC-DHs in adult male or female rat or mice models of CIPN or PDN even for long-term durations [82, 83, 99, 104].

It has become clear that there are great discrepancies even for the minimal infiltration of T or αβ T cells into the SC-DHs after nerve injuries. This may arise from the differences in animal genetic backgrounds or immune conditioning during the life histories before nerve injuries [92, 96, 105–109] and the intrinsic distinctions of nerve injuries [96, 108, 110, 111]. However, chronic tactile allodynia was still significantly developed in all the conditions of nerve injuries. Therefore, the negligible infiltration of T cells and even αβ T cells may be a stochastic but not causal process against nerve injuries. In another word, it seems that the SC-DH is at least not an indispensable neuroimmune interface for T cells and even αβ T cells, not to mention Th cells, to initiate the chronification of tactile allodynia after nerve injuries.

Currently, it has been shown that the infiltration of T cells (CD3 positive) into the injured nerves is a general and intrinsic process in response to rodent or human mechanical or non-mechanical nerve injuries [99, 100, 112, 113]. However, there are contrasting evidences showing that CD3-positive T cells do not significantly infiltrated into the injured nerves in adult C57BL/6 J male or female mice of CIPN or PDN [82, 99]. To go further, αβ T cells have been conclusively found to infiltrate into the injured nerves after CCI or chronic mild compression and acute crush to the sciatic nerves of adult rat or mice [80, 102, 103, 105, 111, 114]. We have also shown that after adult rat TNT, αβ T cells robustly infiltrate into the injured tibial nerves, rather than the intact sural nerves or the glabrous skin tissues innervated by either the sural or tibial nerves [15]. It is worth noting that CD3-positive T cells are significantly increased in the paw skin 8 and 28 days after SNI in in adult C57BL/6 male and female mice and 8 weeks after streptozotocin-mediated diabetic induction in adult C57BL/6 female but not male mice [87, 99]. The infiltration of αβ T cells into injured nerves remains to be examined in more nerve injury conditions, such as SSNL and PSNL. However, it remains uncertain whether these αβ T cells are CD4^+^ αβ T cells and even MHCII-restricted Th cells. Recently, CD4^+^ cells were shown to robustly infiltrate into the injured nerves after adult mice CCI or PSNL [115, 116], and the infiltration of CD4^+^ cells into chronically constricted sciatic nerves are dependent on MHCII specifically expressed on Schwann cells [115]. This suggests the potential infiltration of CD4^+^ αβ T cells and even Th cells into the injured nerves, which needs further validations.

It should be questioned whether αβ T cells and even possible Th cells infiltrating into the injured nerves take part in the development of chronic tactile allodynia after nerve injuries. We have used *prior* lymphadenectomy to popliteal or sciatic lymph nodes for region-specific targeting of αβ T cells in injured tibial nerves and demonstrated the dispensability of these T cells for the development of chronic mechanical allodynia after adult rat TNT [15]. Hence, the injured nerves are not a necessary neuroimmune interface for αβ T cells, including possible Th cells, to initiate the development of chronic tactile allodynia after nerve injuries. This notion needs further validation via region-specific cell targeting in a different set of nerve injury models, such as CCI, in which intact peripheral axons of primary sensory neurons (PSNs) transmitting tactile allodynia are mixed with degenerating peripheral axons of axotomized PSNs [115–117]. However, the number of αβ T cells in the injured nerves was shown to have no correlation with the intensity of tactile allodynia after adult rat CCI to the sciatic nerve [105]. This might suggest a dispensable role of αβ T cells and even possible Th cells in partially injured nerves for the transition to chronic tactile allodynia.

The parenchyma of dorsal root ganglia (pDRGs) have also been suggested as a potential neuroimmune interface for T cells to initiate the transition to chronic tactile allodynia after nerve injuries [87, 102]. Previous studies by other groups indicated that T cells (CD3 positive or Lck-zsGreen positive) are significantly present in the pDRGs from 7 to 28 days after SNI or PSNL in adult male or female C57BL/6 mice [84, 87, 99, 100, 112]. Moreover, CD3-positive T cells and even CD4-positive T cells were shown to be increased in the pDRGs 7 days after low-dose paclitaxel induction in adult male C57BL/6 J mice [83, 118], while no significant changes were seen for the number of CD3-positive T cells in the pDRGs 13 days after high-dose paclitaxel induction in adult male C57BL/6 J mice [82]. CD3-positive T cells were also found to be increased 8 weeks in adult female C57BL/6 J mice but 19 weeks in adult female C57BL/6 J mice after streptozotocin-mediated diabetic induction [99, 119].

However, in our previous study, 7 days after TNT in adult male SD rats, very few, if any, αβ T cells were observed in the pDRGs [15]. This suggested that T cells infiltrating into the cell body-rich areas of DRGs would be largely αβ TCR-negative T cells, i.e., γδ T cells. In fact, the development of T cells in Lck-Cre transgenic mice is biased to γδ T cells [120]. In agreement, only a very small number of αβ T cells were found to significantly infiltrate into the pDRGs after CCI or chronic mild compression to the sciatic nerves as well as L5 SSNL in adult rats [96, 102, 103, 105, 108, 111]. Moreover, in the pDRGs, the presence or absence of αβ T cells [111] and even the number of αβ T cells in the case of significant αβ T cell infiltration [101, 103, 105] do not correlate with the development of chronic tactile allodynia after nerve injuries. Hence, it could be concluded that the pDRGs are at least not the necessary neuroimmune interface for αβ T cells (including Th cells) to initiate the development of chronic tactile allodynia after nerve injuries.

In summary, current evidences suggest that SC-DHs, injured nerves, and pDRGs are not the reasonable pathogenic neuroimmune interfaces for Th cells to light up chronic tactile allodynia after nerve injuries. However, there is no solid and direct evidence for either the infiltration of Th cells into these three classical neuroimmune interfaces or functional implications of potential Th cell infiltration in the chronification of tactile allodynia after nerve injuries (Fig. 7a) [15]. Current evidences are derived from just the study of T or αβ T cell infiltration patterns, with no specific intention to the complexity of T cell heterogeneity and region-specific functional validation. One of the most prominent reasons is the lack of the purposeful and adequate use of accurate and efficient methods to identify or trace Th cell infiltration after nerve injuries and to target potential infiltrating Th cells in a region-specific manner.

### Dorsal root leptomeninge (DRLM) as a novel pathogenic neuroimmune interface

In our previous study, we found, for the first time, that CD4^+^CD8^−^ αβ T cells (presumably Th cells) robustly and selectively infiltrate into the leptomeninges across the whole courses of the lumbar dorsal roots (DRs) along the neuroaxis after nerve injuries (Fig. 7a–c) [15]. This is in consistent with a series of seminal studies, which suggested that after nerve injuries, αβ T cells robustly infiltrate into the leptomeninges of the subarachnoid angles (SAAs) at the transitional zone between the lumbar DRs and DRGs [96, 108, 110]. However, whether these CD4^+^ αβ T cells are Th cells needs further validation by excluding the potential existence of innate CD4^+^ αβ T cells. To establish the universality, this phenomenon should also be further characterized with different conditions of nerve injuries in different species. However, the difficulties in accurate and effective harvest and staining of DRLMs make this task technically challenging (Fig. 7b) [15].

From the point of view of anatomy, the dorsal root (DR) meninges are the lateral extension of spinal meninges and presumably have the same embryonic origin as the spinal meninges (Fig. 7b) [15, 121, 122]. This is further supported by the histological similarity of immunological elements in DR and cerebrospinal meninges (Fig. 7b) [15]. In detail, there are MHCII-expressing macrophages and DCs, which line on the DRLMs adjacent to the CSF compartments [122, 123]. In the intervertebral foramens, lymphatic vessels are present in the DR dura mater and epidural tissues [124]. There is also a rich amount of blood microvessels in the DRLMs [122, 125, 126]. Given the anatomical, embryonic, and histological similarities, the DR meninges are likely to be functionally similar, if not identical, to cerebrospinal meninges for Th cell interactions with the nervous system [58].

Following this suggestion, our noteworthy study provides the first evidence that DRLM CD4^+^ αβ T cells (presumably Th cells) contribute to the transition from acute to chronic tactile allodynia after nerve injuries [15]. In brief, *prior* lymphadenectomy to lumbar lymph nodes (LLNs), where CD4^+^ αβ T cells in DRLMs are derived (Fig. 7b), specifically reduces the development of chronic tactile allodynia after nerve injuries. More importantly, intrathecal application of the suppressive anti-αβ TCR antibodies, which specifically depleted DRLM CD4^+^ αβ T cells, reduces the development of nerve injury-induced chronic tactile allodynia (Fig. 7b, d). This functional validation would be further strengthened by additional rescue experiments, i.e., orthotropic adoptive transfer of nerve injury-conditioned, isolated Th cells into Th cell lacking animals.

## Directions for future research-an interdisciplinary perspective

With certain notable limitations for the current state of preclinical and clinical evidences, MHCII-restricted Th cells have been clearly shown as an important trigger for chronic tactile allodynia after nerve injuries. Moreover, the definite pathogenic neuroimmune interfaces for these Th cells remain controversial, not to mention the detailed mechanisms. One of the most important reasons underlying this dispirited state is that studies on neuroimmune interactions in chronic neuropathic pain (including Th cells in chronic tactile allodynia) are, more often than not, designed by and for neuroscientists themselves. The neglected and inadequate adoption of immunological perspective, nomenclature, and techniques makes the current evidences inconsistent. Therefore, we discuss below that further studies with an interdisciplinary perspective, Th cell neuroimmunology in particular, will provide a more comprehensive understanding of Th cells as a trigger for chronic tactile allodynia after nerve injuries.

First, it is fundamental to ascertain, at more detailed and extended temporal scales, where along the neuroaxis Th cells infiltrate to act as a trigger for chronic tactile allodynia after diverse nerve injuries across different animal species. This will provide the anatomical basis for further mechanistic studies. Technically, further studies should use accurate and efficient Th cell identification/tracing techniques and region-specific Th cell targeting techniques. As mentioned above, the most accurate identification of Th cells requires combinatorial use of CD4/αβ TCR or CD4/CD3 double labeling as Th cell detection method in the form of histology in situ or flow cytometry and MHCII knockout mice as Th cell depletion method. For technical practice, PLZF would be used as a relatively accurate biomarker to distinguish Th cells from innate CD4^+^ αβ T cells. Moreover, the use of transgenic fluorescent reporter mice for Th cell lineage members (including T cell, αβ T cell, and CD4^+^ T cell) [84] and whole mount/body labeling and imaging [127–129] will facilitate the mapping of Th cell infiltration along the neuroaxis after nerve injuries, especially for the delicate DRLMs. Last but not the least, region-specific pharmacological depletion of Th cells [15] and orthotropic adoptive transfer of nerve injury-conditioned, isolated Th cells into MHCII knockout mice, will aid in the validation of the definite pathogenic neuroimmune interface for Th cells to take part in the transition to chronic tactile allodynia after nerve injuries.

Second, it remains to uncover how Th cells acts as a trigger for chronic tactile allodynia after nerve injuries. If DRLMs, as we reported, were the definite pathogenic neuroimmune interface for Th cells, we need to know what the detailed molecular phenotype for these pathogenic Th cells is, how they infiltrate there, and how they execute their pathogenic effects on the neuroaxis in spite of their absence in the neuroaxial parenchyma. The phenotyping of pathogenic Th cells lies in the center of these questions. The complexity of Th cell polarization renders doubtful the traditional methods that use immunohistochemistry and flow cytometry with the selected but limited molecular markers to define a particular subset or state of Th cells [55]. Single-cell transcriptomics has been increasingly used to phenotype the subsets or states of immune cells, including Th cells [56, 57]. The molecular landscape of these pathogenic Th cells at single-cell resolution will aid in clarifying how they infiltrate into DRLMs and fulfill their pathogenic role. Another important question to determine is whether the pathogenic role of Th cells here is dependent on MHCII, i.e., antigen specific [130]. In fact, several studies have suggested antigen specificity for these pathogenic Th cells [81, 95, 97, 103]. If this proves to be true in the future, the successful mapping of the cognate antigens will aid in deeper insights into the pathogenic mechanisms of Th cells and therefore translate into the development of antigen-specific immunoprevention of chronic tactile allodynia after nerve injuries.

Last, it remains to uncover why Th cells can act as a trigger for chronic tactile allodynia after nerve injuries in susceptible individuals. MHCII gene polymorphism has been shown to be associated with the risk of chronic tactile allodynia after nerve injuries [69]. However, it remains unknown how the disease-predisposing MHCII haplotypes result in the break of immune tolerance to self-antigens, i.e., the production of pathogenic autoimmune Th cells after nerve injuries. The knowledge about central or peripheral immune tolerance and MHCII gene polymorphism in the pathogenesis of organ-specific autoimmune diseases, such as type I diabetes or multiple sclerosis, would provide key paradigms in terms of immunological perspectives and techniques [131, 132]. It is worthy to note, however, that disease-predisposing MHCII haplotypes do not always result in the corresponding autoimmune diseases [62, 69]. In fact, the complex interaction between particular genes, genomic backgrounds, and epigenomic states determines the genetic penetrance of disease-predisposing genes, including MHCII here, in the development of autoimmune diseases [133]. Elucidating this multidimensional interaction in the development of chronic tactile allodynia after nerve injuries will aid in the development of a composite biomarker signature to effectively stratify prospective patients for whom Th cells acts as a trigger for chronic tactile allodynia after nerve injuries [134]. This will facilitate the prevention and treatment of chronic tactile allodynia after nerve injuries by targeting autoimmune Th cells from the perspective of precision medicine.

## Conclusions

Elucidating why and how individuals develop or withstand chronic pain after nerve injuries will pave the way for the development of new therapeutic strategies in the context of precision medicine to either prevent or reverse chronic peripheral neuropathic pain. A growing body of clinical and preclinical evidences has clearly showed that Th cells act as an important trigger for chronic tactile allodynia after nerve injuries in some susceptible individuals. Therefore, for translation pain research, an increased intention should be paid to the detailed roles and mechanisms for Th cells in the transition to chronic tactile allodynia after nerve injuries.

Current studies designed by and for neuroscientists themselves have suffered from the neglected and inadequate adoption of immunological perspective, nomenclature, and techniques of Th cells. Here, we introduce a comprehensive interdisciplinary perspective, Th cell neuroimmunology in particular, for neuroscientists who desire deeper insights into pain neuroimmunology. With the state-of-art methods for studying Th cells available in time, future studies with an interdisciplinary perspective will ultimately provide a more comprehensive understanding of whether, where, how, and why Th cells do during the development of chronic tactile allodynia after nerve injuries. This will lay down the foundation for the prevention and treatment of chronic tactile allodynia after nerve injuries by targeting autoimmune Th cells from the perspective of precision medicine.

## Data Availability

Not applicable.

## Abbreviations

AICD: Activation-induced cell death
APC: Antigen-presenting cell
BBB: Blood-brain barrier
CCI: chronic constriction injury
CCK_2_: Cholecystokinin type 2 receptor
CIPN: Chemotherapy-induced peripheral neuropathy
CNS: Central nervous system
CPA: Conditioned place aversion
CRPS: Complex regional pain syndrome
CSF: Cerebrospinal fluid
cTEC: Cortical thymic epithelial cell
CTS: Carpal tunnel syndrome
DC: Dendritic cell
DH: Dorsal horn
DP: Double positive
DR: Dorsal root
DRLM: Dorsal root leptomeninge
EAE: Experimental autoimmune encephalomyelitis
FRC: Fibroblastic reticular cell
fTreg: Follicular Treg
GABA: γ-Aminobutyric acid
GBS: Guillain-Barre syndrome
HLA: Human leukocyte antigen
iTreg: Inducible Treg
LDH: Lumbar disc herniation
LLN: Lumbar lymph node
LTMR: Low-threshold mechanoreceptor
MCP-3: Monocyte chemotactic protein 3
MHCII: Major histocompatibility complex class II
MOR: Mu opioid receptor
mTEC: Mmedullary thymic epithelial cell
NK: Natural killer
NKT: Natural killer T cell
PDN: Painful diabetic neuropathy
pDRG: Parenchyma of dorsal root ganglia
PHN: Postherpetic neuralgia
PLZF: Promyelocytic leukemia zinc finger
pMHCII: Peptide-MHCII
PNS: Peripheral nervous system
PSN: Primary sensory neuron
PSNL: Partial sciatic nerve ligation
PTN: Pain transmission neuron
PV: Parvalbumin
RVM: Rostral ventromedial medulla
SAA: Subarachnoid angle
SC: Spinal cord
SC-DH: Spinal cord dorsal horn
SD: *Sprague-Dawley*
SNI: Spared nerve injury
SP: Single positive
SSNL: Selective spinal nerve ligation
TCR: T cell receptor
TFH: Follicular T helper
TGN: Trigeminal neuralgia
Th: T helper
TLT: Tertiary lymphoid tissue
TNT: Tibial nerve transection
TRA: Tissue-restricted antigen
UEA: Ubiquitous-expressed antigen
VFH: von Frey hair
